# Text Mining-Based Drug Discovery for Connective Tissue Disease–Associated Pulmonary Arterial Hypertension

**DOI:** 10.3389/fphar.2022.743210

**Published:** 2022-03-18

**Authors:** Jiang-Shan Tan, Song Hu, Ting-Ting Guo, Lu Hua, Xiao-Jian Wang

**Affiliations:** ^1^ Key Laboratory of Pulmonary Vascular Medicine, State Key Laboratory of Cardiovascular Disease, Center for Respiratory and Pulmonary Vascular Diseases, National Clinical Research Center of Cardiovascular Diseases, National Center for Cardiovascular Diseases, Department of Cardiology, Fuwai Hospital, Chinese Academy of Medical Sciences and Peking Union Medical College, Beijing, China; ^2^ Key Laboratory of Pulmonary Vascular Medicine, State Key Laboratory of Cardiovascular Disease, Center for Respiratory and Pulmonary Vascular Diseases, National Center for Cardiovascular Diseases, Fuwai Hospital, Chinese Academy of Medical Sciences and Peking Union Medical College, Beijing, China

**Keywords:** text mining, connective tissue disease, pulmonary arterial hypertension, drug discovery, drugs

## Abstract

**Background:** The current medical treatments for connective tissue disease–associated pulmonary arterial hypertension (CTD-PAH) do not show favorable efficiency for all patients, and identification of novel drugs is desired.

**Methods:** Text mining was performed to obtain CTD- and PAH-related gene sets, and the intersection of the two gene sets was analyzed for functional enrichment through DAVID. The protein–protein interaction network of the overlapping genes and the significant gene modules were determined using STRING. The enriched candidate genes were further analyzed by Drug Gene Interaction database to identify drugs with potential therapeutic effects on CTD-PAH.

**Results:** Based on text mining analysis, 179 genes related to CTD and PAH were identified. Through enrichment analysis of the genes, 20 genes representing six pathways were obtained. To further narrow the scope of potential existing drugs, we selected targeted drugs with a Query Score ≥5 and Interaction Score ≥1. Finally, 13 drugs targeting the six genes were selected as candidate drugs, which were divided into four drug–gene interaction types, and 12 of them had initial drug indications approved by the FDA. The potential gene targets of the drugs on this list are IL-6 (one drug) and IL-1β (two drugs), MMP9 (one drug), VEGFA (three drugs), TGFB1 (one drug), and EGFR (five drugs). These drugs might be used to treat CTD-PAH.

**Conclusion:** We identified 13 drugs targeting six genes that may have potential therapeutic effects on CTD-PAH.

## Introduction

Pulmonary arterial hypertension (PAH) is a life-threatening complication of connective tissue disease (CTD) and has been described in patients with systemic sclerosis (SSc), systemic lupus erythematosus (SLE), mixed CTD (MCTD), primary Sjögren’s syndrome, and rheumatoid arthritis (Sung and Chung, 2015). In Western countries, CTD-associated pulmonary arterial hypertension (CTD-PAH) was the second leading cause of PAH (25%), and almost 75% of CTD-PAH was SSc-related; the 3-year survival within this population was only 56% (Hachulla et al., 2009; Thakkar and Lau, 2016). Similarly, CTD-PAH was the third most common type of PAH (20%) in China (Jiang et al., 2012), and more than half of these cases were SLE-related (58.4%) (Zhao et al., 2017). The estimated 1-year and 3-years survival rates were 85.4 and 53.6%, respectively (Zhang et al., 2011). Recently, PAH-targeted drugs were used in patients with CTD-PAH, but the effect was unsatisfactory (Fisher et al., 2006; Zhang et al., 2011). Immunosuppressive therapy is an indispensable part of treatment for CTD-PAH as well (Kato et al., 2020). The administration of immunosuppressive agents, including systemic glucocorticoids and intravenous cyclophosphamide, may improve the clinical status of PAH patients who have SLE, MCTD, and Sjögren’s syndrome; however, patients with SSc-PAH do not respond well to immunosuppressive therapy (Sanchez et al., 2006; Kato and Atsumi, 2018; Kato et al., 2020).

As mentioned above, an optimal regimen of treatment has not been established, and existing drugs do not show favorable efficiency for all CTD-PAH patients; therefore, novel drugs for this condition are needed. Drug discovery has long been a time- and capital-intensive process, and an unpredictable return on investment may result. Nevertheless, drug repurposing serves as a quicker, and a less costly method to expand indications for already approved drugs (Moosavinasab et al., 2016). A successful example is Pfizer’s sildenafil, which was originally designed for the treatment of angina but then repositioned to treat erectile dysfunction in 1998 (Novac, 2013). Text mining is a feasible strategy to discover drugs with new treatment potential (Andronis et al., 2011). Based on the available literature and biomedical databases, combined with analytical tools, text mining aims to explore drugs with potential value for the treatment of targeted diseases among the currently available drugs. In this study, we discovered that some existing drugs may be used for CTD-PAH treatment by text mining.

## Materials and Methods

### Text Mining

Text mining was performed to obtain disease–gene associations automatically based on a substantial number of biological studies, which was performed by querying the pubmed2ensembl database though http://pubmed2ensembl.ls.manchester.ac.uk/. The pubmed2ensembl is an extension of the BioMart system, which contains more than 2,000,000 articles in PubMed and almost 150,000 genes in Ensembl (Baran et al., 2011). With pubmed2ensembl, we can extract all of the associated genes using search strings from the available biological literature when we perform a query. In the present study, we performed two queries: one with the concept “pulmonary arterial hypertension” (PAH) and another with the concept “connective tissue diseases” (CTD). All unique genes were extracted from each result. Then, we conducted a genetic screen to identify intersecting genes that participate in both PAH and CTD.

### Gene Ontology and Pathway Enrichment Analysis

The Gene Ontology (GO) analysis includes three main branches: cellular component (CC), molecular function (MF), and biological process (BP). The Kyoto Encyclopedia of Genes and Genomes (KEGG) (Kanehisa and Goto, 2000) is an open access and systematic analysis database from Japan that specializes in annotation and pathway enrichment analysis. The GO analysis and KEGG enrichment analysis of candidate overlapping genes were performed with DAVID (https://david.ncifcrf.gov/), an online gene functional annotation tool with visualization and gene attributes. A *p* value <0.05 was required for statistical significance as the threshold.

### Protein Interaction and Module Analysis

We performed an analysis on the protein–protein interaction (PPI) network of the candidate overlapping genes using the web-based tool STRING (version 11.0, http://string-db.org/) (Szklarczyk et al., 2015). First, the overlapping genes were uploaded into the STRING website with a significance threshold of a minimum interaction score >0.9 (high confidence). Then, the TSV format file of PPI was downloaded, and further analysis was performed with Cytoscape software. STRING and the Molecular Complex Detection (MCODE) app were built in Cytoscape and used to classify the significant gene modules (clusters). The parameters in MCODE were set by default. Finally, the drug–gene interaction analysis was performed based on the genes in the gene modules.

### Drug–Gene Interactions

The Drug Gene Interaction database (http://www.dgidb.org) was used for further analysis of drug–gene interactions based on the final list of genes, which could be used as potential therapeutic targets in a search for existing drugs (Wagner et al., 2016). Due to a large number of predicted drugs, we chose the targeted drugs with stringent criteria: Query Score ≥5 and Interaction Score ≥1. These candidate drugs targeting the genes/pathways relevant to PAH and CTD may represent potential treatments.

## Results

### Results of Text Mining, GO, and Pathway Enrichment Analysis

**FIGURE 1:**
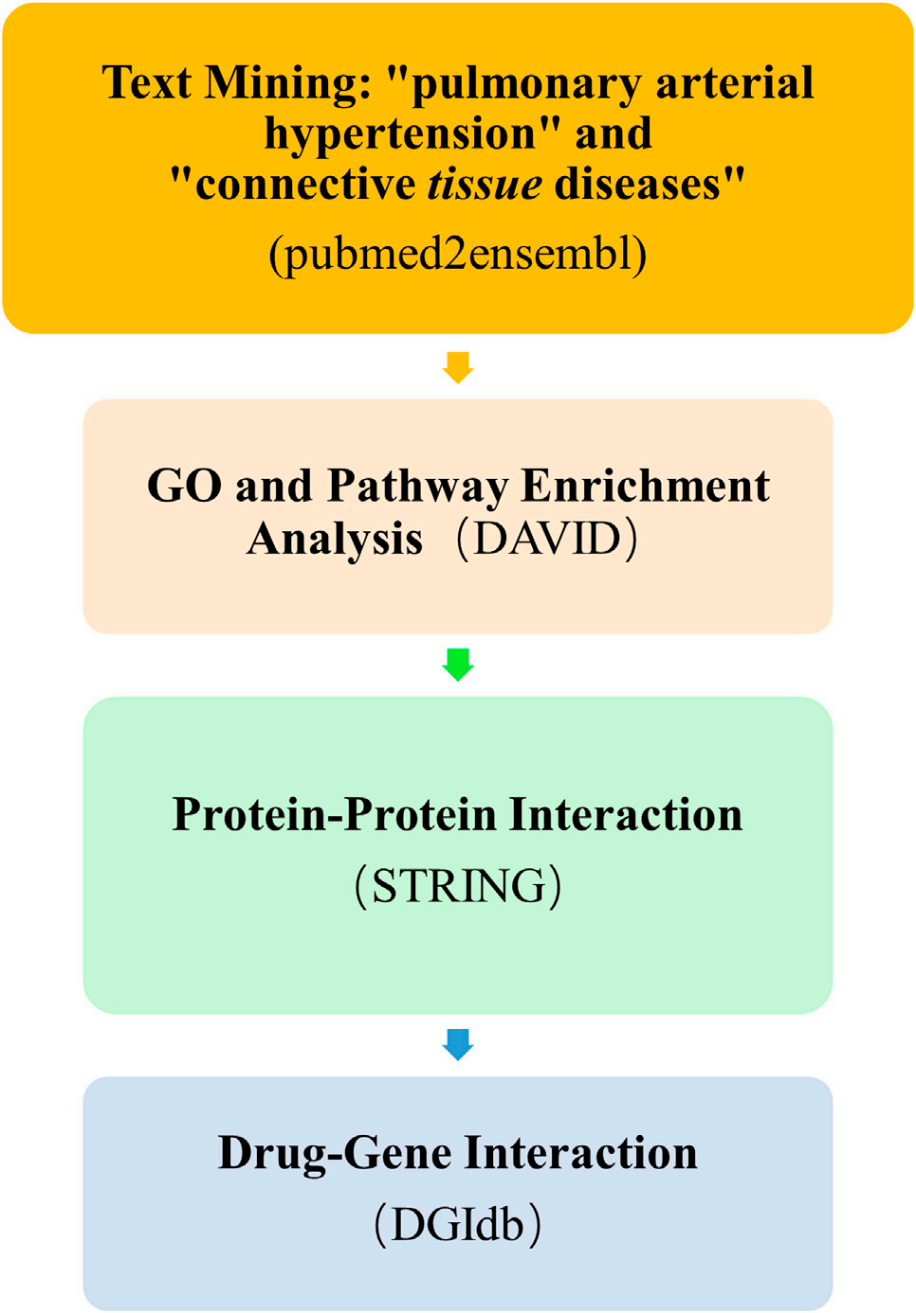
Overall text mining strategy. pubmed2ensembl was used to identify connective tissue disease–associated pulmonary arterial hypertension (CTD-PAH)–related gene sets, and the overlapping genes of the two gene sets were analyzed for enrichment by DAVID. The protein–protein interaction network of the overlapping genes and the enriched candidate genes were shown using STRING. The final enriched candidate genes for further analysis of drug–gene interactions by DGIdb to determine dugs with potential therapeutic effects of CTD-PAH. GO, Gene Ontology; DGIdb, Drug Gene Interaction database.

**FIGURE 2:**
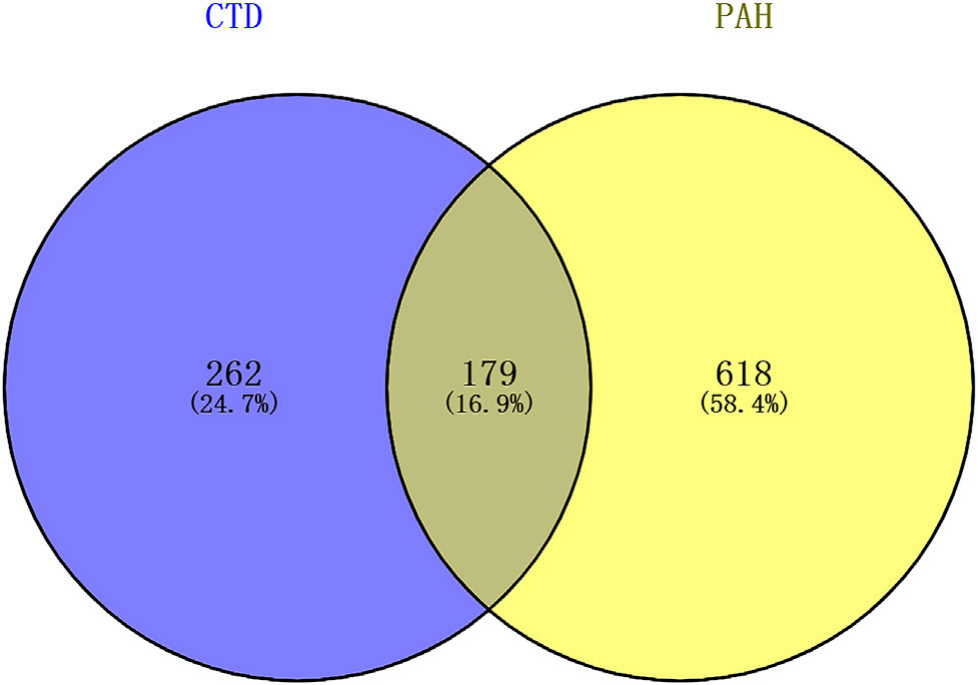
The results of text mining. A total of 797 genes were related to PAH, 441 genes were related to CTD, and 179 genes overlapped between PAH and CTD. PAH, pulmonary arterial hypertension; CTD, connective tissue diseases.

**FIGURE 3:**
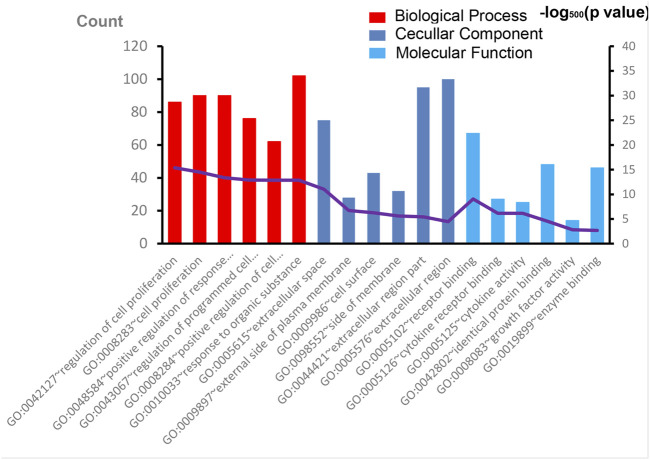
The results of GO enrichment analysis. The top six significant enrichment terms of biological process, cellular component, and molecular function of overlapping genes are shown. GO, Gene Ontology.

**FIGURE 4:**
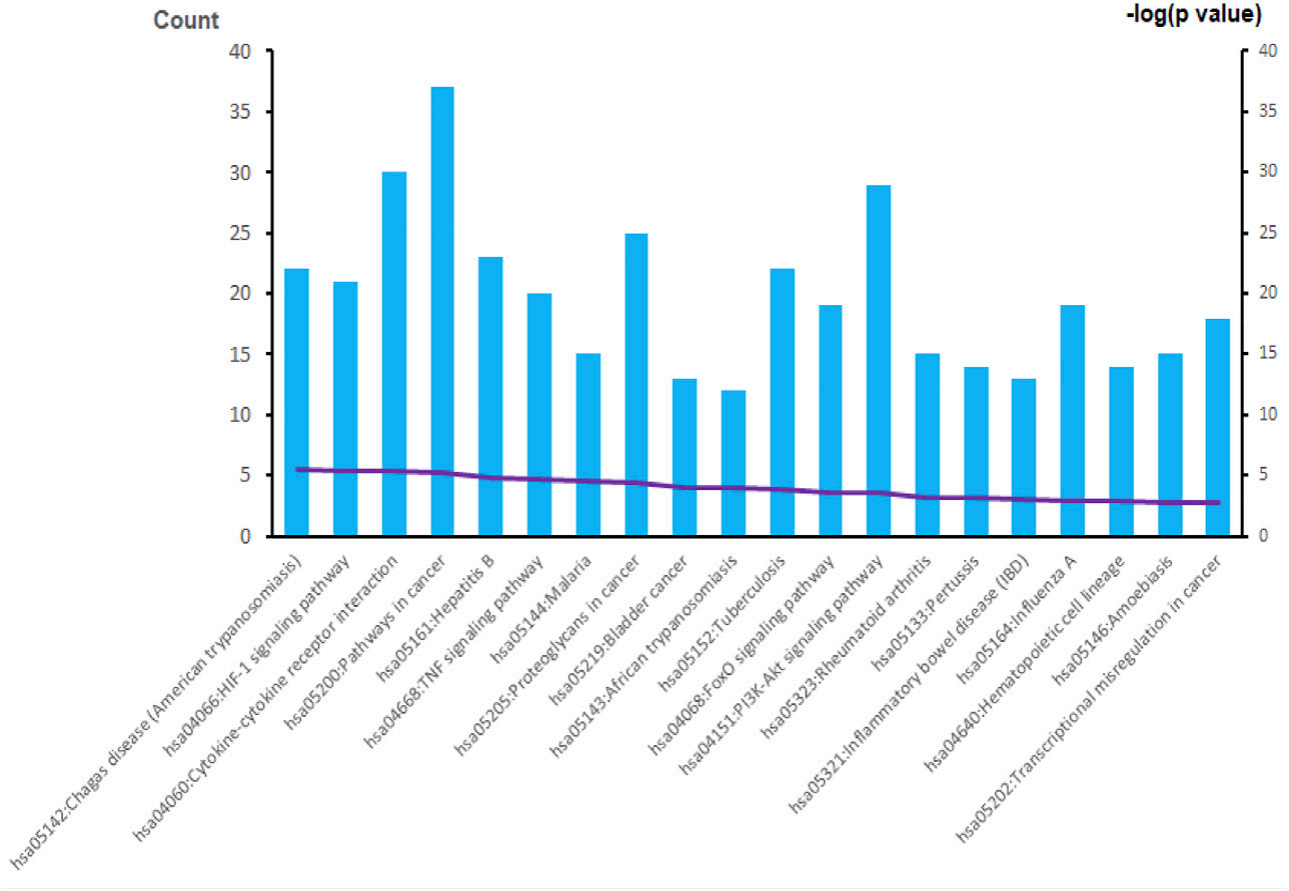
The results of KEGG enrichment analysis. A total of 20 KEGG signaling pathways of overlapping genes are shown. KEGG, Kyoto Encyclopedia of Genes and Genomes.

The overall data mining strategy is described in Figure 1. From text mining searches, 797 genes were related to PAH, 441 genes were related to CTD, and 179 genes overlapped between PAH and CTD (Figure 2). The GO analysis was classified into three functional categories: BP, CC, and MF (in Figure 3, the top six significant enrichment terms for BP, CC, and MF and the top 20 KEGG signal pathways of the overlapping genes are shown). In the BP term, GO analysis showed that these common genes were mainly enriched in the regulation of response to organic substance, cell proliferation, and positive regulation of response to stimulus. In the CC term, these common genes were significantly enriched in the extracellular region, extracellular region part, and extracellular space. In the MF term, these common genes were mainly enriched in receptor binding, identical protein binding, and enzyme binding. Signaling pathway enrichment showed that these common genes were mainly involved in cancer, cytokine–cytokine receptor interaction, and the PI3K-Akt signaling pathway (Figure 4).

### Protein Interaction and Module Analysis

**FIGURE 5:**
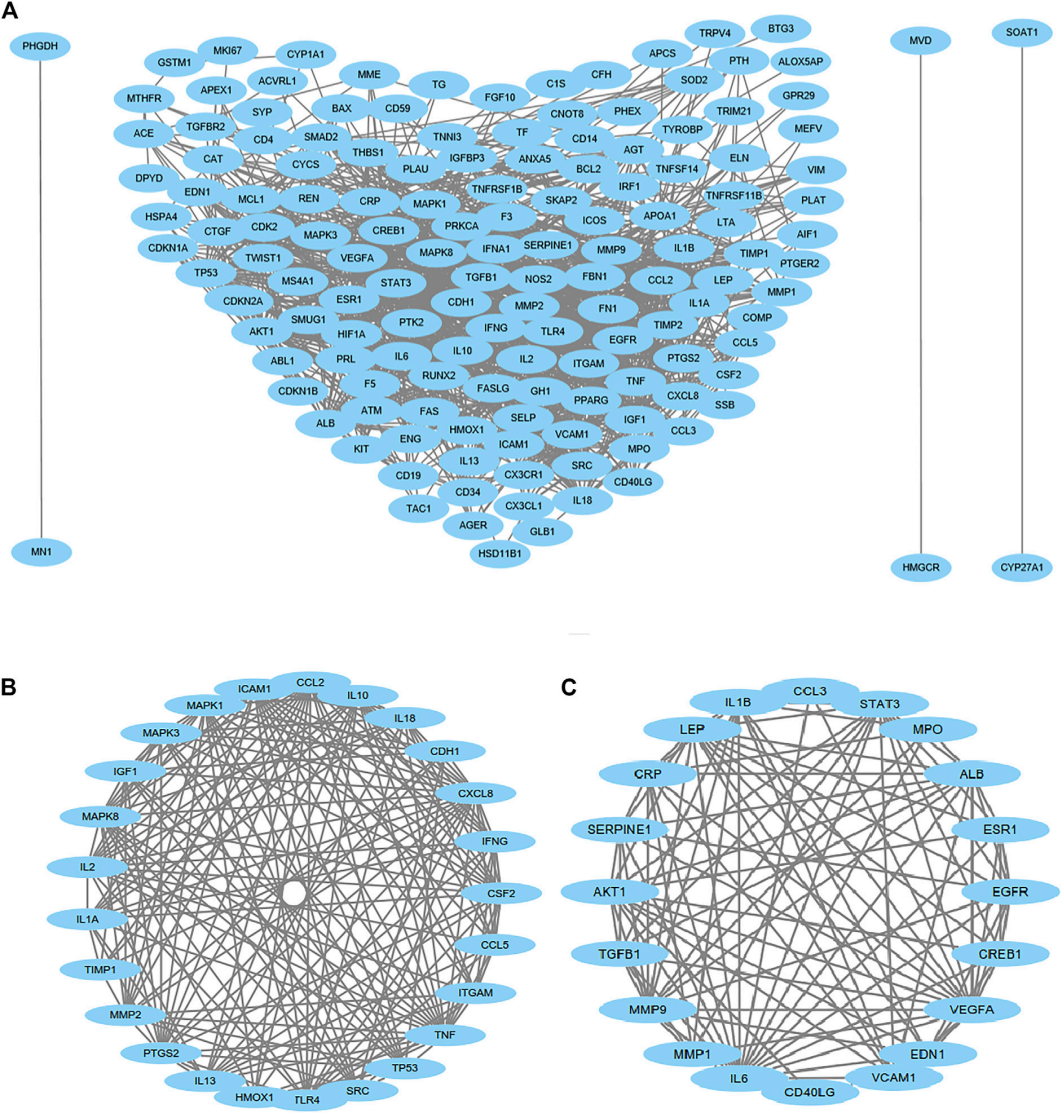
Protein–protein interaction (PPI) network analysis. PPI of 149 genes/nodes with scores >0.9 (high confidence) and 1,205 edges are shown in **(A)**, and 30 genes have been excluded because of low confidence. For the most significant gene modules, two significant gene modules were clustered by using the MCODE application. Module 1 consisted of 25 genes/nodes and 180 edges **(B)**, and module 2 was made up of 20 genes/nodes and 104 edges **(C)**.

We then uploaded the 179 genes to the STRING website to construct the PPI networks. After excluding 30 genes with low confidence (score <0.9), we identified 149 genes/nodes with score >0.9 (high confidence) and 1,205 edges in the construction of the PPI networks (Figure 5A). By using the MCODE application, we clustered two significant gene modules. Module 1 consisted of 25 genes/nodes and 180 edges (Figure 5B), while module 2 was made up of 20 genes/nodes and 104 edges (Figure 5C).

### Drug–Gene Interaction and Functional Analysis of Potential Genes

To simplify our module and identify high-efficiency drugs to treat CTD-PAH, we chose module 2 for further analysis. The 20 genes in module 2 were selected for drug–gene interaction analysis. A total of 12 genes were targeted by 76 potential existing drugs, which were divided into 28 drug–gene interaction types, and all had initial drug indications (Supplementary Table S1). The potential gene targets of the drugs on this list are SERPINE1, IL6, IL1B, MMP9, MPO, VEGFA, VCAM1, ALB, ESR1, CREB1, TGFB1, and EGFR. Almost half of the drugs (36 of the 76) target estrogen receptor alpha (ESR1) and interact with estrogen receptor alpha in an inhibitory or antagonistic manner (Supplementary Table S1). To further narrow the scope of the potential existing drugs, we selected targeted drugs with Query Score ≥5 and Interaction Score ≥1 in the final results (Table 1). A total of six genes were targeted by 13 potential existing drugs, which were divided into four drug–gene interaction types, and 12 of them had initial drug indications approved by the FDA (Table 1). The potential gene targets of the drugs on this list are IL6 (one drug), IL1B (two drugs), MMP9 (one drug), VEGFA (three drugs), TGFB1 (one drug), and EGFR (five drugs).

**TABLE 1:** The available drugs that can target six candidate genes.

ID	Drug	Gene	Interaction types	Administration	Approved use by the FDA	Sources	Query score	Interaction score	PubMed ID
1	Siltuximab	IL6	Antagonist|antibody|inhibitor	Intravenous	Multicentric Castleman’s disease	DrugBank|MyCancerGenome	21.78	8.61	88,23,310
2	Canakinumab	IL1B	Inhibitor|binder|antibody	Subcutaneous	Periodic fever syndromes; active Still’s disease	DrugBank|MyCancerGenome	30.49	8.46	19,169,963
3	Rilonacept	IL1B	Binder|inhibitor	Subcutaneous	Cryopyrin-associated periodic syndrome; recurrent pericarditis	DrugBank|ChemblInteractions	7.26	2.01	23,319,019
4	Glucosamine*	MMP9	Antagonist	Oral	—	DrugBank	10.45	2.96	12,405,690
5	Ranibizumab	VEGFA	Inhibitor	Ophthalmic	Neovascular (wet) age-related macular degeneration; macular edema following retinal vein occlusion; diabetic macular edema; diabetic retinopathy; myopic choroidal neovascularization	DrugBank|TdgClinicalTrial	23.96	6.51	18,046,235
6	Pegaptanib Sodium	VEGFA	Antagonist	Ophthalmic	Neovascular (wet) age-related macular degeneration	TdgClinicalTrial|ChemblInteractions	8.71	2.37	23,953,100
7	Aflibercept	VEGFA	Antibody|binder|inhibitor	Ophthalmic	Neovascular (wet) age-related macular degeneration; macular edema following retinal vein occlusion; diabetic macular edema; Diabetic retinopathy	DrugBank|MyCancerGenome	6.97	1.89	22,813,448
8	Hyaluronidase	TGFB1	Inhibitor	Subcutaneous	As an adjuvant	DrugBank	21.78	7.1	9,435,505
9	Afatinib	EGFR	Inhibitor	Oral	Non–small-cell lung cancer	DrugBank|FDA	57.5	4.14	26,619,011
10	Osimertinib	EGFR	Inhibitor	Oral	Non–small-cell lung cancer	DrugBank|FDA	31.36	2.26	31,825,714
11	Dacomitinib	EGFR	Inhibitor	Oral	Non–small-cell lung cancer	DrugBankCKB|FDA	26.13	1.88	24,857,124
12	Necitumumab	EGFR	Antagonist|inhibitor|antibody	Intravenous	Non–small-cell lung cancer	TALC|DrugBank	17.42	1.26	20,197,484
13	Erlotinib	EGFR	Antagonist|inhibitor	Oral	Non–small-cell lung cancer; Pancreatic cancer	DrugBank|FDA	14.63	1.05	26,619,011

FDA, Food and Drug Administration.

*This drug has not yet been approved by the FDA (indicated use being investigated).

## Discussion

CTD-PAH shares similar pathophysiological characteristics with other types of PAH, and dysfunction of multiple cells and molecular processes may contribute to vasoconstriction and inflammation of arterioles, which results in progressive increases in pulmonary vascular resistance and right ventricular afterload, heart failure, and even death (Thenappan et al., 2018b). Currently, all PAH-targeted drugs mainly target pulmonary vasoconstriction rather than vascular remodeling, which underlies the basic pathological characteristics of PAH. By performing text mining, we found six genes of interest and 13 potential drugs for the treatment of CTD-PAH. Notably, the main drivers of vascular remodeling, including endothelial cells (ECs) or pulmonary artery smooth muscle cells (PASMCs), were implicated in the identified target gene–related signaling pathway.

Immunological disturbance and inflammatory mechanisms play a central role during the development of PAH in CTD (Dorfmüller et al., 2003). Elevated serum levels of some proinflammatory cytokines, such as interleukin (IL)-1 and IL-6, were reported in CTD-PAH patients (Dorfmüller et al., 2003). The downregulation of IL-1β and IL-6 expressions could inhibit the development of PAH by suppressing the proliferation and migration of PASMCs in rats (Ou et al., 2020). A study also demonstrated that the expression of IL-6 was increased in the lung, and IL-6 blockade by the monoclonal anti–IL-6 receptor antibody MR16-1 could ameliorate the pulmonary hypertension (PH) of pristane/hypoxia mice (a novel mouse model of PH reflecting the pathological features of CTD-PAH) (Mori et al., 2020). The IL-6 signaling pathway is a promising candidate in the treatment of CTD-PAH, and the use of tocilizumab, an anti-IL-6 receptor antibody was proven to result in clinical improvements in some cases of CTD-PAH (Arita et al., 2010; Furuya et al., 2010; Kadavath et al., 2014). Although siltuximab (targeting IL-6) seems to have a similar effect on CTD-PAH, the related evidence is limited. As for IL-1β blockade, whether it has a positive effect on CTD-PAH requires more evidence.

Pulmonary vascular remodeling is initiated by remodeling of the extracellular matrix resulting from the imbalance of proteolytic enzymes and their inhibitors (Thenappan et al., 2018a). Remodeling of the ECM of the pulmonary artery occurs in the early stage of PAH pathogenesis and even precedes the hemodynamic changes in pulmonary circulation (Thenappan et al., 2018a). As an important type of proteolytic enzyme, matrix metalloproteinase (MMP) shows an increase in activity associated with the proliferation and migration of PASMCs and intimal thickening during the development of PAH (Chelladurai et al., 2012). In the MMP family, the upregulation of gelatinases (MMP-2 and MMP-9) was identified in monocrotaline (MCT)–induced or hypoxia-induced mouse model of PH, and gelatinases were considered possible therapeutic targets for the treatment of PAH (George and D'Armiento, 2011; Liu et al., 2018). We found the possible use of glucosamine, an antagonist of MMP9, in CTD-PAH, but most studies have focused on its clinical use in patients with osteoarthritis thus far. Moreover, contraindications and drug interactions involving glucosamine use are uncommon (Dahmer and Schiller, 2008; Bruyère et al., 2016), therefore, the possible use of glucosamine should be explored.

The potential effect of epidermal growth factor receptor (EGFR)–mediated survival of PASMCs has led researchers to test the efficacy of selective targeting EGFR in the PH animal model (Merklinger et al., 2005). Dahal et al. (2010) found that erlotinib (one of the first generation EGFR tyrosine kinase inhibitors; it is also the drug we suggested in Table 1) showed therapeutic benefit in rats with MCT-induced PH, but it did not show therapeutic efficacy in chronic hypoxic mice model. The reason why partial therapeutic benefit was observed may be because different mechanisms were involved in these two commonly used animal models with respect to the involvement of EGF signaling. Most importantly, multiple growth factors, including EGF, platelet-derived growth factor, and fibroblast growth factor, are all involved in PASMC proliferation in response to hypoxia. Therefore, no significant therapeutic efficacy was observed in chronic hypoxic mice. Notably, no significant alteration of EGFR expression in the lung tissues from patients with idiopathic PAH was also reported (Dahal et al., 2010). In Dahal’s study, only patients with advanced-stage idiopathic PAH were enrolled. Therefore, as described in their study, the important role of EGF signaling in the early stage of PAH development could not be excluded. However, the second generation of EGFR tyrosine kinase inhibitors and a new pan-EGFR inhibitor, dacomitinib, showed a significant inhibitory effect on pulmonary vascular remodeling and attenuated pulmonary artery pressure and right ventricular hypertrophy in both MCT and hypoxia-induced mice model of PH (Yu et al., 2019). Other drugs that target EGFR in our list (necitumumab, afatinib, and osimertinib) have not yet been validated. The therapeutic effect of EGFR, a promising target, is required to be elucidated in the future.

Disturbed production of vasoactive, vasoconstrictive, and proliferative mediators from ECs may affect vascular tone and promote vascular remodeling (Zanatta et al., 2019). The vascular endothelial growth factor (VEGF) is the main angiogenic factor and is indispensable for the process of normal angiogenesis. The overexpression of VEGF is associated with proliferation of ECs in severe PAH (Sakao and Tatsumi, 2011). VEGF and its receptors showed increased expressions in animal models (hypoxia or MCT-induced PH), and elevated levels of plasma VEGF were also discovered in PAH patients (Voelkel and Gomez-Arroyo, 2014). However, many studies have revealed that combined with a second hit (high shear stress or chronic hypoxia), VEGF receptor blockade can drive the emergence of apoptosis-resistant ECs with the potential for hyperproliferation, and angio-obliterative PAH may form as a result (Sakao et al., 2009; Voelkel and Gomez-Arroyo, 2014). VEGFA is the most abundant isomer of VEGF in humans and is currently the main target of anti-VEGF treatment. It is unknown whether VEGF receptor antagonists would contribute to the development of PAH, but two patients developed PH were reported in patients who had ovarian cancer received bevacizumab (recombinant humanized monoclonal antibody against VEGF-A) (Garcia et al., 2008). Therefore, VEGF receptor antagonists may not be applicable in the treatment of PAH.

The predominant isoform of transforming growth factor β (TGFβ) in humans is TGFβ-1, and excessive TGFβ-1 signaling is a characteristic of PAH (Sturrock et al., 2006; Kajdaniuk et al., 2013; Calvier et al., 2019). By reducing the expression of PTEN and increasing the activation of PI3K/AKT, TGFβ-1 promotes the proliferation and decreases the apoptosis of PASMCs in lung tissue (Liu et al., 2016). Apoptosis-resistant PASMCs could promote progressive narrowing of the vascular lumen and excessive accumulation of ECM components, which reduced vascular compliance (Thenappan et al., 2018b). Selective trapping of the TGFβ-1 ligand has been demonstrated to improve hemodynamics, pulmonary vessel remodeling, and survival in mice model (Yung et al., 2016). Although hyaluronidase can target TGFβ-1, the indication is as an adjuvant to increase the absorption of other drugs in local use (Table 1). Moreover, hyaluronidase cannot be delivered to the lung by the venous system because the enzyme is rapidly inactivated following intravenous administration. Thus, hyaluronidase is not applicable in the clinical treatment of PAH, but a suitable formulation for delivery may be an alternative consideration.

The first limitation of this study is related to the databases we used. Not all the drug–gene interactions are fully clarified in the present databases. Therefore, the analysis may be more accurate as the databases are updated. Additionally, some candidate genes for targeting might have generalized effects that may not always be desirable. Thus, lack of experiments to validate the targets and drugs that we found is the other limitation of the present study. Experiments and clinical trials are required before these drugs are approved for clinical use.

As discussed above, some drugs that we found (Table 2) may have unfavorable effects depending on the current findings. For example, drugs that target VEGFA may induce PAH, erlotinib (target EGFR) was ineffective in animal model, and hyaluronidase (target TGFβ-1) could not be delivered to lung tissue in its present formulation. With the evolution and improvement of database and analytic tools, this method of data mining will promote drug discovery in CTD-PAH therapy.

**TABLE 2:** Comprehensive recommendation of promising drugs for the treatment of CTD-PAH.

Gene targets	Reported in CTD animal model	Reported in PAH animal model	Proposed drugs by text-mining	Tested in clinical trials for CTD or PAH	Recommendation (candidate for therapy of CTD-PAH)	References (PubMed ID)
IL6	Yes	Yes	Siltuximab	No	High possibility	32,522,898
IL1B	Yes	Yes	Canakinumab; Rilonacept	No	High possibility	32,712,318
MMP9	Yes	Yes	Glucosamine	No	High possibility	21,063,214
VEGFA	Yes	Yes	Ranibizumab; Pegaptanib Sodium; Aflibercept	No	Uncertain (the drugs targeting VEGFA may induce PAH)	24,932,885
TGFB1	NO	Yes	Hyaluronidase	No	Less possibility (it could not be delivered to the lungs in its present formulation)	27,115,515
EGFR	Yes	Yes	Afatinib; Osimertinib; Dacomitinib; Necitumumab; Erlotinib	No	High possibility (but ineffective result of Erlotinib was reported in an animal model)	30,753,867

CTD, connective tissue disease; PAH, pulmonary arterial hypertension; CTD-PAH, connective tissue disease–associated pulmonary arterial hypertension.

In conclusion, 13 drugs targeting six genes that may have potential therapeutic effects on CTD-PAH were discovered, and none of them has been tested in clinical trials for PAH patients. Drug discovery by performing text mining and pathway analysis can help identify existing drugs that have the potential to treat CTD-PAH.

## Data Availability

The original contributions presented in the study are included in the article/Supplementary Material, further inquiries can be directed to the corresponding authors.
