# Supplementary material for: Text Mining-Based Drug Discovery for Connective Tissue Disease–Associated Pulmonary Arterial Hypertension
Source: Front Pharmacol. 2022 Mar 18;13:743210. doi: 10.3389/fphar.2022.743210 (PMC8971927; doi:10.3389/fphar.2022.743210)
Supplement: Supplementary file 1 [file Table1.DOCX]

**Supplementary Table 1. Seventy-six potential drugs for twelve gene targets.**

| **ID** | **Drug** | **Gene** | **Interaction types** | **Sources** | **Query Score** | **Interaction Score** | **PubMed ID** |
| --- | --- | --- | --- | --- | --- | --- | --- |
| 1 | UROKINASE | SERPINE1 | inducer\|substrate | DrugBank\|TdgClinicalTrial | 3.05 | 1.47 | 12709915 |
| 2 | SILTUXIMAB | IL6 | antagonist\|antibody\|inhibitor | DrugBank\|MyCancerGenome | 21.78 | 8.61 | 8823310 |
| 3 | MINOCYCLINE | IL1B | modulator | DrugBank | 3.84 | 0.36 | 8943052 |
| 4 | DONEPEZIL | IL1B | inducer\|inhibitor | DrugBank | 1.34 | 0.37 | 20153342 |
| 5 | GALLIUM NITRATE | IL1B | antagonist | DrugBank | 1.24 | 0.35 | 16122880 |
| 6 | RILONACEPT | IL1B | binder\|inhibitor | DrugBank\|ChemblInteractions | 7.26 | 2.01 | 23319019 |
| 7 | CANAKINUMAB | IL1B | inhibitor\|binder\|antibody | DrugBank\|MyCancerGenome | 30.49 | 8.46 | 19169963 |
| 8 | ZINC CHLORIDE | MMP9 | binder | DrugBank | 0.18 | 0.05 | 17137715 |
| 9 | GLUCOSAMINE | MMP9 | antagonist | DrugBank | 10.45 | 2.96 | 12405690 |
| 10 | MINOCYCLINE | MMP9 | inhibitor | DrugBank | 4.61 | 0.87 | 15353401 |
| 11 | CAPTOPRIL | MMP9 | inhibitor | DrugBank | 2.56 | 0.73 | 12381651 |
| 12 | MELATONIN | MPO | inhibitor | DrugBank | 1.02 | 0.13 | 18237195 |
| 13 | CEFDINIR | MPO | inhibitor | DrugBank | 1.74 | 0.87 | 8133056 |
| 14 | CARVEDILOL | VEGFA | other/unknown | DrugBank | 2.04 | 0.18 | 15071347 |
| 15 | RANIBIZUMAB | VEGFA | inhibitor | DrugBank\|TdgClinicalTrial | 23.96 | 6.51 | 18046235 |
| 16 | GLICLAZIDE | VEGFA | other/unknown | DrugBank | 3.11 | 0.85 | 17602961 |
| 17 | PEGAPTANIB SODIUM | VEGFA | antagonist | TdgClinicalTrial\|PharmGKB | 8.71 | 2.37 | 23953100 |
| 18 | AFLIBERCEPT | VEGFA | antibody\|binder\|inhibitor | DrugBank\|MyCancerGenome | 6.97 | 1.89 | 22813448 |
| 19 | MINOCYCLINE | VEGFA | inhibitor | DrugBank | 3.07 | 0.28 | 11875741 |
| 20 | BEVACIZUMAB | VEGFA | inhibitor\|antibody | TALC\|DrugBank | 8.92 | 0.61 | 18182667 |
| 21 | CARVEDILOL | VCAM1 | inhibitor | DrugBank | 1.63 | 0.89 | 17139284 |
| 22 | ARTENIMOL | ALB | ligand | DrugBank | 0.16 | 0.07 | 8347159 |
| 23 | LASOFOXIFENE | ESR1 | negative modulator\|antagonist\|agonist | DrugBank\|TdgClinicalTrial | 7.26 | 0.38 | 17456742 |
| 24 | ESTRIOL | ESR1 | agonist | DrugBank\|TdgClinicalTrial | 7.62 | 0.4 | 2011412 |
| 25 | METHYLDOPA | ESR1 | cofactor | DrugBank | 0.11 | 0 | 14640689 |
| 26 | OSPEMIFENE | ESR1 | agonist\|antagonist\|modulator | DrugBank\|TdgClinicalTrial | 6.53 | 0.34 | 15255284 |
| 27 | NORGESTIMATE | ESR1 | agonist | DrugBank\|TdgClinicalTrial | 6.1 | 0.32 | 14672731 |
| 28 | CLOMIPHENE | ESR1 | agonist\|antagonist | DrugBank\|TdgClinicalTrial | 15.25 | 0.79 | 19761360 |
| 29 | ESTRADIOL | ESR1 | agonist | DTC\|DrugBank | 2.43 | 0.13 | 20334368 |
| 30 | RALOXIFENE | ESR1 | antagonist\|agonist | DTC\|DrugBank | 5.17 | 0.13 | 10477535 |
| 31 | FULVESTRANT | ESR1 | antagonist | TALC\|DTC\|DrugBank | 9.64 | 0.5 | 20151846 |
| 32 | TESTOSTERONE | ESR1 | inhibitor | DTC\|DrugBank | 1.18 | 0.03 | 16038533 |
| 33 | ETHYNODIOL DIACETATE | ESR1 | agonist | DrugBank | 4.36 | 0.23 | 4123746 |
| 34 | MELATONIN | ESR1 | antagonist | DrugBank | 1.54 | 0.02 | 15229223 |
| 35 | NALOXONE | ESR1 | other/unknown\|antagonist | DrugBank | 1.16 | 0.03 | 16546975 |
| 36 | TAMOXIFEN | ESR1 | agonist\|antagonist | DTC\|FDA | 4.49 | 0.08 | 24398047 |
| 37 | ETHINYL ESTRADIOL | ESR1 | agonist | DrugBank\|ChemblInteractions | 3.69 | 0.19 | 17124377 |
| 38 | FLUOXYMESTERONE | ESR1 | antagonist | DrugBank | 5.23 | 0.27 | 3058238 |
| 39 | PRASTERONE | ESR1 | binder | DrugBank | 0.34 | 0.02 | 15994348 |
| 40 | DIENESTROL | ESR1 | agonist | DTC\|DrugBank | 7.08 | 0.37 | 15276617 |
| 41 | ZINC CHLORIDE | ESR1 | binder | DrugBank | 0.18 | 0 | 14640689 |
| 42 | MESTRANOL | ESR1 | agonist | DrugBank\|TdgClinicalTrial | 3.87 | 0.2 | 20497523 |
| 43 | CHLOROTRIANISENE | ESR1 | agonist | DTC\|DrugBank | 7.84 | 0.41 | 6872937 |
| 44 | LEVONORGESTREL | ESR1 | other/unknown | DrugBank\|TdgClinicalTrial | 7.99 | 0.41 | 12589940 |
| 45 | PHENOLPHTHALEIN | ESR1 | agonist | DTC\|DrugBank | 1.09 | 0.06 | 3620717 |
| 46 | TRILOSTANE | ESR1 | allosteric modulator | DrugBank | 2.49 | 0.13 | 16806905 |
| 47 | ALLYLESTRENOL | ESR1 | agonist | DrugBank | 15.25 | 0.79 | 8820988 |
| 48 | ESTROPIPATE | ESR1 | agonist | DrugBank | 7.62 | 0.4 | 17138652 |
| 49 | DANAZOL | ESR1 | agonist | DTC\|DrugBank | 2.31 | 0.12 | 7540578 |
| 50 | ESTRADIOL BENZOATE | ESR1 | agonist | DrugBank | 1.68 | 0.09 | 17138652 |
| 51 | ESTRONE | ESR1 | agonist | DTC\|DrugBank | 3.96 | 0.21 | 2011412 |
| 52 | QUINESTROL | ESR1 | modulator\|agonist | DrugBank\|TdgClinicalTrial | 7.84 | 0.41 | 11752352 |
| 53 | BAZEDOXIFENE | ESR1 | agonist\|antagonist | DrugBank\|TdgClinicalTrial | 5.44 | 0.28 | 15535430 |
| 54 | PROGESTERONE | ESR1 | negative modulator\|inhibitor\|agonist | DrugBank\|TdgClinicalTrial | 0.62 | 0.03 | 26153859 |
| 55 | TOREMIFENE | ESR1 | modulator | DrugBank\|FDA | 18.51 | 0.48 | 12870885 |
| 56 | DESOGESTREL | ESR1 | agonist | DrugBank\|TdgClinicalTrial | 6.53 | 0.34 | 10836199 |
| 57 | TIBOLONE | ESR1 | antagonist\|agonist | DrugBank\|GuideToPharmacology | 4.36 | 0.23 | 19464167 |
| 58 | ETONOGESTREL | ESR1 | agonist | TdgClinicalTrial\|TEND | 4.36 | 0.23 | 17139284 |
| 59 | NALOXONE | CREB1 | other/unknown | DrugBank | 3.48 | 3.79 | 12969258 |
| 60 | TERAZOSIN | TGFB1 | inducer | DrugBank | 0.93 | 0.3 | 23518907 |
| 61 | HYALURONIDASE | TGFB1 | inhibitor | DrugBank | 21.78 | 7.1 | 9435505 |
| 62 | IBRUTINIB | EGFR | inhibitor | DTC\|GuideToPharmacology | 0.57 | 0.04 | 24915291 |
| 63 | NECITUMUMAB | EGFR | antagonist\|inhibitor\|antibody | TALC\|DrugBank | 17.42 | 1.26 | 20197484 |
| 64 | PANITUMUMAB | EGFR | suppressor\|antibody\|inhibitor | TALC\|FDA | 10.16 | 0.73 | 17355997 |
| 65 | VANDETANIB | EGFR | inhibitor | TALC\|DrugBank | 4.28 | 0.15 | 18681783 |
| 66 | BRIGATINIB | EGFR | inhibitor | DrugBank\|ChemblInteractions | 3.48 | 0.25 | 23239810 |
| 67 | GEFITINIB | EGFR | antagonist\|inhibitor | DrugBank\|FDA | 8.97 | 0.65 | 24533047 |
| 68 | CETUXIMAB | EGFR | antagonist\|inhibitor\|antibody | TALC\|FDA | 19.73 | 0.71 | 26619011 |
| 69 | NERATINIB | EGFR | inhibitor | DrugBank\|PharmGKB | 22.17 | 0.8 | 18681783 |
| 70 | FOSTAMATINIB | EGFR | inhibitor | DrugBank | 0.03 | 0 | 26516587 |
| 71 | DACOMITINIB | EGFR | inhibitor | DrugBank\|FDA | 26.13 | 1.88 | 24857124 |
| 72 | LIDOCAINE | EGFR | antagonist | DrugBank\|TdgClinicalTrial | 1.45 | 0.05 | 16551906 |
| 73 | AFATINIB | EGFR | inhibitor | DrugBankCKB\|FDA | 57.5 | 4.14 | 26619011 |
| 74 | LAPATINIB | EGFR | inhibitor\|antagonist | DTC\|DrugBankCKB | 15.25 | 0.55 | 25305330 |
| 75 | OSIMERTINIB | EGFR | inhibitor | DrugBank\|FDA | 31.36 | 2.26 | 31825714 |
| 76 | ERLOTINIB | EGFR | antagonist\|inhibitor | DrugBank\|FDA | 14.63 | 1.05 | 26619011 |
